# Limited Discriminatory Capacity of Continuous Glucose Monitoring in Prediabetes and Increased Diabetes Risk: A Cross-Sectional Study

**DOI:** 10.3390/medicina62081539

**Published:** 2026-08-11

**Authors:** Dóra Marietta Balogh, Karola Osgyán, Magdolna Békeffy, Miklós Kempler, Gergely Balaton, Manfredi Rizzo, Viktor József Horváth, Péter Kempler, Adrienn Menyhárt

**Affiliations:** 1 Department of Internal Medicine and Oncology, Semmelweis University, Korányi Sándor u. 2/a., 1083 Budapest, Hungary; baloghd90@gmail.com (D.M.B.); osgyankarola@gmail.com (K.O.); bekeffy.magdi95@gmail.com (M.B.);; 2 Department of Internal Medicine and Haematology, Semmelweis University, Szentkirályi u. 46., 1088 Budapest, Hungary; kemplersoma@gmail.com; 3 Department of Paediatric Dentistry and Orthodontics, Semmelweis University, Szentkirályi u. 47., 1088 Budapest, Hungary; gergelybalaton@gmail.com; 4 School of Medicine, ProMISE Department, University of Palermo, 90133 Palermo, Italy; manfredi.rizzo@unipa.it

**Keywords:** FINDRISC, prediabetes, continuous glucose monitoring, HbA1c

## Abstract

*Background and Objectives:* To determine whether continuous glucose monitoring (CGM)-derived metrics can differentiate between normoglycemia, increased diabetes risk (FINDRISC+) and prediabetes and to assess whether CGM-derived metrics provide additional discriminatory information beyond conventional laboratory markers. *Materials and Methods:* In this cross-sectional study, 41 adults without diabetes were classified as normoglycemic controls (HbA1c < 5.7%, FINDRISC < 12), individuals at increased diabetes risk (HbA1c < 5.7%, FINDRISC ≥ 12), or individuals with prediabetes (HbA1c 5.7–6.49%). All participants underwent 14-day CGM. Mean interstitial glucose, glycemic variability (SD, CV) and time in, above and below range (TIR, TAR, TBR) were analyzed. Group differences were assessed using ANOVA or non-parametric tests; correlations were evaluated using Pearson analysis. *Results:* HbA1c differed significantly across groups (F = 17.62; *p* < 0.001). Among CGM metrics, only mean interstitial glucose differed (F = 3.54; *p* = 0.039), driven by higher values in the prediabetes group. Glycemic variability and time-based metrics did not differ significantly. Substantial overlap was observed between the increased risk and prediabetes groups. No significant correlation was found between HbA1c and mean CGM glucose (r = 0.164; *p* = 0.30). *Conclusions:* In this exploratory cohort, short-term CGM-derived metrics provided limited additional discriminatory information beyond conventional laboratory markers and clinical risk assessment. Larger prospective studies are needed to determine the discriminatory and predictive value of CGM in early metabolic risk states.

## 1. Introduction

The prevention and early detection of diabetes mellitus are of paramount importance, as complications associated with disturbances in glucose metabolism may develop even before the onset of overt disease [1,2]. Prediabetes is defined as an intermediate state of hyperglycemia, characterized by glucose levels above normal but below the diagnostic threshold for diabetes [3]. In addition to prediabetes, increasing attention has been directed toward individuals who, despite having normoglycemia based on laboratory parameters, are at elevated risk of developing diabetes due to clinical and lifestyle-related factors. The Finnish Diabetes Risk Score (FINDRISC) questionnaire is a widely used and validated tool for identifying and estimating this risk [4]. Risk assessment tools such as FINDRISC identify individuals at increased risk based on clinical and lifestyle-related factors rather than direct biochemical evidence of dysglycemia. Large population-based studies, including the METSIM study, have demonstrated that clinical risk scores are strongly associated with insulin resistance, impaired insulin secretion, incident diabetes, cardiovascular risk and mortality, highlighting that metabolic risk extends well beyond glycemic biomarkers alone [5,6].

In recent years, continuous glucose monitoring (CGM) has gained increasing importance not only in the management of diabetes mellitus but also in the assessment of glucose-related metabolic states [7]. CGM enables detailed characterization of short-term glycemic exposure, mean glucose levels and glycemic variability, using parameters such as standard deviation (SD), coefficient of variation (CV) and time spent in, above, and below the target range (TIR, TAR, TBR). Although CGM has become an integral component of diabetes management, its clinical role in individuals with prediabetes or increased diabetes risk remains considerably less well established. While several studies in individuals with prediabetes have demonstrated that CGM-derived metrics reflect a less favorable glucose profile compared with normoglycemic controls, including higher mean glucose levels and increased glycemic variability [8,9], it remains uncertain whether CGM is sufficiently sensitive to detect more subtle alterations in glucose regulation in earlier, preclinical stages, particularly in individuals who are at increased risk but do not yet exhibit biochemical evidence of dysglycemia.

However, limited data are available on whether CGM profiles differ between individuals with increased diabetes risk but preserved normoglycemia (e.g., those with high FINDRISC scores) and those with prediabetes [10,11]. This distinction is clinically relevant, as risk-based stratification and biochemically defined prediabetes often identify overlapping but not identical populations, which may require different preventive and monitoring strategies. Furthermore, it remains unclear whether CGM-derived parameters are capable of discriminating between these early metabolic states, and to what extent short-term glycemic patterns reflect longer-term metabolic risk—a question with direct implications for early detection and targeted prevention.

Based on these considerations, the aim of our study was to determine whether CGM-derived parameters can differentiate normoglycemic control individuals from those at increased diabetes risk (high FINDRISC score) and from individuals with prediabetes. Furthermore, we assessed whether CGM-derived glucose profiles provide additional discriminatory information beyond conventional laboratory markers in the early stages of dysglycemia.

## 2. Materials and Methods

### 2.1. Subjects

The overall study period was between 1 March and 30 September 2025, at the Department of Internal Medicine and Oncology, Semmelweis University, Budapest, Hungary. Before ethics approval was obtained on 10 March 2025, only preliminary recruitment activities, including contacting potential participants to assess their willingness to participate and arranging study appointments, were carried out. All study-specific procedures, including written informed consent, CGM placement, and research data collection, were initiated only after ethics approval had been obtained. Adults aged ≥18 years under outpatient care at our institution were eligible for inclusion. The study was carried out in accordance with the Declaration of Helsinki and applicable national regulations. All participants provided written informed consent prior to enrollment. The study protocol was approved by the National Institute of Public Health and Pharmacology (approval number: NNGYK/07600-4/2025). Exclusion criteria included any history of diabetes mellitus, chronic kidney disease (KDIGO stage IIIb–V), untreated thyroid disease, systemic autoimmune disorders, New York Heart Association class II–IV heart failure, acute infection or hospitalization within 30 days prior to enrollment.

### 2.2. Clinical and Metabolic Assessment

Participants attended a study visit after an overnight fast of at least 12 h. Demographic data (age, sex) and clinical information, including hypertension, hyperlipidemia, smoking status and family history of diabetes, were recorded. Body weight (in light clothing) and height (without shoes) were measured using calibrated instruments and body mass index (BMI) was calculated as kg/m^2^. Waist circumference was measured in the standing position, 2 cm above the umbilicus. Blood pressure was measured on the left arm in a seated position after at least 5 min of rest and the mean of two measurements was recorded using an automated device (Omron M2; OMRON Healthcare Co., Ltd., Kyoto, Japan). Venous blood samples were obtained from the antecubital vein. Serum glucose, HbA1c, total cholesterol, LDL cholesterol, HDL cholesterol and triglycerides were analyzed in the central laboratory of our institution using standard methods.

### 2.3. Definition of Metabolic Status

Participants were classified into three groups based on HbA1c levels and Finnish Diabetes Risk Score (FINDRISC). Normoglycemic controls were defined as individuals with HbA1c < 5.7% and FINDRISC < 12. Individuals at increased diabetes risk (FINDRISC+) were defined as those with HbA1c < 5.7% and FINDRISC ≥ 12. Prediabetes was defined as HbA1c 5.7–6.49%, irrespective of FINDRISC score. The term “increased diabetes risk (FINDRISC+)” was used to describe individuals with elevated clinical risk in the absence of biochemical dysglycemia, distinguishing this group from biochemically defined prediabetes. The FINDRISC questionnaire was administered using the validated Hungarian provided by the Hungarian Diabetes Association.

### 2.4. Continuous Glucose Monitoring

Following clinical and laboratory assessments, participants underwent continuous glucose monitoring (CGM) using the CareSens Air system (i-SENS, Inc., Seoul, Republic of Korea; distributed in Hungary by 77 Elektronika Ltd., Budapest, Hungary). The CareSens Air CGM system has demonstrated good analytical performance in previous validation studies, with an overall mean absolute relative difference (MARD) of 10.42% and 99.92% of paired measurements falling within Consensus Error Grid zones A and B [12]. The sensor was placed on the posterior upper arm according to the manufacturer’s instructions. Participants received standardized education on device use and were instructed to maintain their usual diet and physical activity throughout the monitoring period. CGM data were collected over 14 consecutive days. After sensor removal, data were downloaded and analyzed.

The following CGM-derived metrics were evaluated in accordance with international recommendations [13,14]: mean interstitial glucose, glucose management indicator (GMI), coefficient of variation (CV), time in range (TIR), time above range (TAR) and time below range (TBR). GMI was calculated using the formula: GMI = 3.31 + 0.02392 × mean interstitial glucose. Normoglycemia was defined as interstitial glucose values between 4.0 and 10.0 mmol/L. Values above this range were classified as TAR and values below as TBR.

### 2.5. Outcome Measures

The primary objective was to assess whether CGM-derived parameters can differentiate between normoglycemic controls, individuals at increased diabetes risk (FINDRISC+) and those with prediabetes. Secondary analyses included the comparison of CGM-derived metrics across groups and the evaluation of the relationship between HbA1c and mean interstitial glucose.

### 2.6. Statistical Analysis

Descriptive statistics were used to summarize clinical, laboratory, and CGM-derived parameters. Continuous variables were presented as mean ± standard deviation (SD) or median with interquartile range, as appropriate. Categorical variables are expressed as counts and percentages. Normality was assessed using the Shapiro–Wilk test, and homogeneity of variances was evaluated with Levene’s test. Differences between the three groups were analyzed using one-way analysis of variance (ANOVA), followed by Tukey post hoc testing when assumptions were met. Effect sizes for one-way ANOVA analyses were quantified using eta squared (η^2^) and interpreted as small (≥0.01), moderate (≥0.06) or large (≥0.14). Cohen’s d was calculated for selected pairwise comparisons. Because of the exploratory nature of the study and the use of an available cohort, no formal a priori sample size calculation was performed. When normality assumptions were violated, the Kruskal–Wallis test was applied. Categorical variables were compared using the chi-square test or Fisher’s exact test, as appropriate. The association between HbA1c and mean interstitial glucose was assessed using Pearson correlation analysis. Because the evaluated CGM-derived metrics represented predefined outcomes rather than exploratory screening variables, no formal adjustment for multiple comparisons was applied. All statistical analyses were performed using SPSS version 27.0 (IBM Corp., Armonk, NY, USA). A two-sided *p* value < 0.05 was considered statistically significant.

## 3. Results

### 3.1. Study Population Characteristics

A total of 41 participants were included in the analysis. Of these, 12 (29%) were classified as normoglycemic controls, 14 (34%) as individuals at increased diabetes risk (FINDRISC+) and 15 (37%) as having prediabetes (HbA1c 5.70–6.49%). Baseline clinical and laboratory characteristics are summarized in Table 1. Mean age differed significantly across groups, with lower values in the control group and higher values in both the increased risk and prediabetes groups. Similarly, body mass index and waist circumference were higher in the two higher-risk groups compared with controls.

Among lipid parameters, HDL cholesterol levels differed significantly, with lower values in the increased risk and prediabetes groups, while total cholesterol, LDL cholesterol and triglycerides did not differ significantly between groups. Sex distribution and blood pressure values were comparable across groups; however, the prevalence of diagnosed hypertension and hyperlipidemia differed significantly.

As expected, HbA1c differed significantly across groups (ANOVA: F = 17.62; *p* < 0.001), with progressively higher values from controls to prediabetes.

### 3.2. Comparison of CGM-Derived Metrics Across Metabolic Groups

CGM-derived metrics stratified by metabolic status are presented in Table 2. Among CGM parameters, only mean interstitial glucose differed significantly across groups (ANOVA: F = 3.54; *p* = 0.039), with all other CGM-derived metrics showing no significant differences. Mean interstitial glucose was numerically higher in the prediabetes group than in the control and FINDRISC+ groups. Although the overall group effect was statistically significant (ANOVA, *p* = 0.039), none of the Tukey-adjusted pairwise comparisons reached statistical significance. Mean interstitial glucose was 5.85 mmol/L (95% CI 5.54–6.16) in controls, 5.85 mmol/L (95% CI 5.57–6.13) in the FINDRISC+ group, and 6.29 mmol/L (95% CI 5.99–6.60) in the prediabetes group.

In contrast, measures of glycemic variability, including standard deviation (SD), coefficient of variation (CV) and time-based metrics (TIR, TAR, TBR), did not differ significantly between groups (all *p* > 0.05). Time in range was high across all groups, whereas TAR and TBR values remained low and showed substantial overlap. Similarly, glucose management indicator (GMI) values did not differ significantly.

Effect-size analysis showed a large overall group effect for mean interstitial glucose (η^2^ = 0.157), whereas the effects for SD (η^2^ = 0.031) and CV (η^2^ = 0.003) were small and negligible, respectively. Pairwise comparisons showed no meaningful difference in mean interstitial glucose between the control and FINDRISC+ groups (Cohen’s d ≈ 0), while large differences were observed between the prediabetes group and both the control and FINDRISC+ groups (Cohen’s d = 0.84 and 0.85, respectively).

Overall, HbA1c discriminated the predefined metabolic groups more clearly than the evaluated CGM-derived metrics. Whereas HbA1c differed significantly across all groups, only mean interstitial glucose showed a significant between-group difference among the CGM-derived parameters, while glycemic variability metrics demonstrated substantial overlap.

### 3.3. CGM Profiles in Increased Diabetes Risk and Prediabetes

Direct comparison of the increased diabetes risk and prediabetes groups revealed substantial overlap in CGM-derived metrics. Although mean interstitial glucose was numerically higher in the prediabetes group, measures of glycemic variability (SD, CV, TIR/TAR/TBR) were comparable between the two groups.

These findings indicate that CGM-derived glucose profiles alone were insufficient to distinguish individuals at increased diabetes risk from those with prediabetes.

### 3.4. Relationship Between HbA1c and Mean CGM Glucose

The correlation between HbA1c and mean interstitial glucose was weak and not statistically significant (Pearson’s r = 0.164; *p* = 0.30) indicating limited agreement between short-term CGM-derived glucose levels and longer-term glycemic exposure.

## 4. Discussion

### 4.1. Principal Findings

The aim of this study was to determine whether CGM-derived parameters can differentiate between normoglycemic individuals, those at increased diabetes risk (FINDRISC+) and individuals with prediabetes. Our findings indicate that most CGM-derived metrics do not clearly distinguish between these metabolic states. Measures of glycemic variability (SD, CV, TIR, TAR, TBR) showed substantial overlap across groups, while only mean interstitial glucose differed modestly, primarily due to higher values in the prediabetes group.

### 4.2. Limited Additional Discriminatory Information of CGM in Early Metabolic States

Previous studies have shown that individuals with prediabetes exhibit less favorable CGM profiles compared with normoglycemic individuals, including higher mean glucose levels and increased glycemic variability [8,9,15]. While our findings are partly consistent with these observations, they also demonstrate that CGM profiles of individuals at increased diabetes risk and those with prediabetes overlap substantially.

In our cohort, no distinct CGM-derived phenotype could be identified that reliably differentiated individuals with increased diabetes risk (FINDRISC+) from those with prediabetes. This finding is clinically relevant, as risk-based classification and biochemically defined prediabetes often identify overlapping but not identical populations, which may require different preventive strategies.

Importantly, individuals with high FINDRISC scores may exhibit completely normal glucose levels, whereas individuals with prediabetes may have low clinical risk scores [16,17]. The observed overlap in CGM-derived glycemic metrics between these groups suggests that short-term glucose patterns may not adequately reflect the complexity of long-term metabolic risk.

In contrast, the FINDRISC score integrates multiple non-glycemic factors, including age, adiposity, family history, and lifestyle, which may better capture the broader metabolic risk profile. This interpretation is supported by large population-based studies, such as the METSIM cohort, demonstrating strong associations between FINDRISC scores and insulin resistance, impaired insulin secretion, cardiovascular risk and mortality [5,18].

### 4.3. HbA1c and CGM Reflect Different Aspects of Glycemia

In our study, HbA1c levels differed more clearly between metabolic groups than CGM-derived metrics, indicating that HbA1c remains a robust discriminator at the group level. However, the weak and non-significant correlation between HbA1c and mean CGM glucose suggests that these measures reflect partially distinct aspects of glycemic exposure.

HbA1c reflects average glycemia over the lifespan of erythrocytes (approximately 8–12 weeks), whereas CGM provides detailed, real-time information on short-term glucose fluctuations over a limited monitoring period. Consequently, CGM-derived metrics may capture dynamic glycemic patterns that are not reflected in HbA1c, while HbA1c integrates longer-term exposure but is insensitive to short-term variability.

Our findings are consistent with previous reports demonstrating weak associations between HbA1c and CGM metrics in non-diabetic or early dysglycemic populations [11]. Rather than indicating that these methods assess fundamentally different aspects of glycemia, this weak association is expected because HbA1c and CGM reflect glycemic exposure over different time scales. HbA1c integrates average glycemia over approximately 8–12 weeks, whereas the present study evaluated glucose profiles during a 14-day monitoring period. Furthermore, biological variability, differences in erythrocyte lifespan, and day-to-day variation in glucose exposure may all contribute to discrepancies between these measures. Taken together, these observations suggest that HbA1c and CGM provide complementary rather than interchangeable information in the assessment of early metabolic dysregulation. These findings are further supported by our recent analysis conducted in the same cohort, which demonstrated that CGM-derived metrics were not associated with early autonomic dysfunction, whereas overall metabolic risk status showed a stronger relationship (Békeffy et al., in press).

It should also be noted that HbA1c may be influenced by non-glycemic factors, including alterations in erythrocyte turnover and hemoglobinopathies [19,20,21], further contributing to discrepancies between HbA1c and CGM-derived measures. Current ADA Standards of Care also acknowledge that HbA1c may differ from CGM-derived mean glucose, particularly in the presence of high glycemic variability or conditions affecting red blood cell lifespan [21]. CGM-derived metrics (including TIR, TAR, TBR, and CV) provide detailed information on daily glycemic patterns and the risk of acute glycemic excursions, which are not captured by HbA1c [19,20,22,23]. In early metabolic states, the association between HbA1c and CGM metrics is typically weak, further supporting that these measures reflect distinct aspects of glycemic physiology [24].

### 4.4. Metabolic Risk Extends Beyond Glycemia

The observed differences in age and HDL cholesterol levels across groups further support the concept that early metabolic risk is not solely defined by glycemic parameters [25]. Individuals at increased diabetes risk and those with prediabetes exhibited a less favorable cardiometabolic profile despite largely overlapping CGM-derived glucose patterns.

Importantly, age should not be regarded simply as a confounding variable in the present study but rather as an integral component of metabolic risk. Similarly, BMI, waist circumference and hypertension constitute key components of the FINDRISC score used to define one of the study groups. Therefore, adjusting for these variables would have removed part of the biological information inherent to the clinical risk classification (over-adjustment) and was not considered appropriate for addressing the primary study objective.

These findings reinforce the notion that short-term glycemic metrics alone may not adequately capture the complexity of early metabolic dysregulation.

### 4.5. Clinical Implications

From a clinical perspective, our results suggest that CGM has limited utility for fine risk stratification in the early metabolic spectrum. Routine use of CGM in individuals with increased diabetes risk may not provide substantial additional information beyond that obtained from established clinical risk scores and conventional laboratory markers. Although current ADA Standards of Care strongly support the use of CGM in individuals with diabetes requiring glucose-lowering therapy, our findings suggest that its routine use for risk stratification in individuals without diabetes provides limited additional discriminatory information beyond established clinical risk scores and conventional laboratory markers [26].

This has potential implications for healthcare resource allocation, as CGM is a relatively resource-intensive tool. In this population, risk assessment based on clinical and laboratory parameters may remain the most practical and informative approach.

However, the present cross-sectional design does not allow conclusions regarding the temporal sequence of metabolic abnormalities. Prospective longitudinal studies are needed to determine whether subtle CGM-derived alterations precede biochemical dysglycemia and whether CGM provides predictive value for future diabetes development. Future studies should also explore whether combining CGM with markers of insulin resistance, inflammatory biomarkers or metabolomic profiling improves the identification of individuals at increased metabolic risk. In addition, longer periods of CGM may better capture habitual glycemic patterns and further clarify the role of CGM in the early stages of metabolic dysregulation. In addition, the evaluation of advanced CGM-derived glycemic variability metrics, such as MAGE or CONGA, may help determine whether these indices provide greater discriminatory capacity than conventional CGM metrics.

### 4.6. Strengths and Limitations

The study has notable strengths, including the well-characterized cohort, the combined use of clinical risk assessment and biochemical classification, a standardized 14-day CGM protocol and the comprehensive evaluation of CGM-derived metrics.

The main limitation of this study is the relatively small sample size, which reduces statistical power and limits the generalizability of the findings. Because of the cross-sectional design, causal relationships cannot be established. In addition, although a 14-day CGM period is consistent with current international recommendations, it may not fully reflect longer-term glycemic variability or habitual glucose patterns. The relatively small sample size may have limited the ability to detect small or moderate between-group differences and increased the possibility of Type II error. Therefore, the findings should be considered exploratory and require confirmation in larger cohorts.

Another limitation is that prediabetes was defined exclusively using HbA1c without oral glucose tolerance testing. Although HbA1c is an accepted diagnostic criterion, it may not identify all individuals with impaired glucose tolerance or isolated impaired fasting glucose. Consequently, some degree of misclassification cannot be excluded. Future studies combining CGM with OGTT-based phenotyping may provide a more comprehensive characterization of early metabolic dysregulation.

Also, dietary intake was not systematically recorded during the CGM period. Although participants were instructed to maintain their usual eating habits, differences in dietary composition and meal timing may have contributed to interindividual variability in CGM-derived metrics. Similarly, physical activity was not objectively monitored and may also have influenced short-term glucose profiles.

## 5. Conclusions

Taken together, our findings indicate that CGM-derived glucose profiles show substantial overlap between normoglycemic individuals, those at increased diabetes risk and individuals with prediabetes. In this exploratory cohort, conventional CGM-derived glycemic variability metrics did not demonstrate meaningful separation among these predefined metabolic groups, whereas mean interstitial glucose differed primarily in participants with prediabetes. These findings suggest that short-term CGM-derived metrics provide limited additional discriminatory information beyond established clinical risk assessment and conventional laboratory markers in the early metabolic spectrum. Larger prospective studies are warranted to determine the discriminatory and predictive value of CGM in early metabolic risk states.

## Figures and Tables

**Table 1:** Baseline clinical and laboratory characteristics of participants according to metabolic status (normoglycemic controls, increased diabetes risk [FINDRISC+] and prediabetes).

	Control (*n* = 12)				Increased Diabetes Risk (*n* = 14)				Prediabetes (*n* = 15)				
	Mean	SD	Lower 95% CI	Upper 95% CI	Mean	SD	Lower 95% CI	Upper 95% CI	Mean	SD	Lower 95% CI	Upper 95% CI	*p*
**Age (years)**	34.2	7.5	30.0	38.4	43.8	11.0	38.1	49.6	57.5	9.0	52.9	62.0	0.001
**Male sex,** ***n*** **(%)**	5 (42%)				7 (50%)				8 (53%)				0.829
**Waist (cm)**	83.5	10.3	77.7	89.3	93.4	8.2	89.1	97.7	96.6	9.2	91.9	101.3	0.003
**BMI (kg/m^2^)**	23.3	2.8	21.8	24.9	26.4	3.1	24.7	28.0	30.0	6.1	26.9	33.0	0.002
**SBP (mmHg)**	123	10	117	129	133	16	124	141	136	9	131	140	0.03
**DBP (mmHg)**	73	8	69	77	78	10	73	83	82	8	78	86	<0.04
**HR (beats/min)**	62	12	55	69	69	11	63	75	65	7	62	69	0.23
**TG (mmol/L)**	0.8	0.3	0.6	1.0	1.5	1.5	0.7	2.3	1.9	1.7	1.0	2.8	0.13
**Chol (mmol/L)**	5.3	0.7	4.9	5.7	5.2	1.1	4.6	5.7	5.5	1.6	4.7	6.3	0.78
**HDL (mmol/L)**	2.0	0.4	1.8	2.2	1.5	0.3	1.4	1.7	1.5	0.5	1.2	1.7	0.002
**LDL (mmol/L)**	3.0	0.6	2.6	3.3	3.2	0.9	2.7	3.7	3.3	1.4	2.6	4.1	0.67
**MIG (mmol/L)**	4.8	0.3	4.6	4.9	4.9	0.4	4.7	5.0	5.3	0.8	4.9	5.7	0.03
**HbA1c (%)**	5.2	0.2	5.1	5.3	5.4	0.2	5.3	5.5	5.8	0.4	5.6	6.0	0.001
**FINDRISK**	2	3	1	4	12	4	10	14	16	5	14	19	0.001
**Hypertension,** ***n*** **(%)**	0 (%)				4 (29%)				9 (60%)				0.004
**Hyperlipidaemia,** ***n*** **(%)**	1 (8%)				4 (29%)				10 (67%)				0.006
**Current smoking,** ***n*** **(%)**	1 (8%)				1 (7%)				0 (0%)				0.101
**Mother DM,** ***n*** **(%)**	1 (8%)				6 (43%)				4 (27%)				0.141
**Father DM,** ***n*** **(%)**	0 (0%)				3 (21%)				7 (47%)				0.019
**Autonomic neuropathy,** ***n*** **(%)**	1 (8%)				7 (50%)				8 (53%)				0.034

Data are presented as mean ± standard deviation or as counts (percentages), as appropriate. Categorical variables are presented as counts and percentages. BMI: body mass index; SBP: systolic blood pressure; DBP: diastolic blood pressure; HR: heart rate; TG: triglycerides; Chol: total cholesterol; HDL: high-density lipoprotein; LDL: low-density lipoprotein; MIG: mean interstitial glucose; FINDRISC: Finnish Diabetes Risk Score.

**Table 2:** Continuous glucose monitoring (CGM)-derived metrics according to metabolic status (normoglycemic controls, increased diabetes risk [FINDRISC+] and prediabetes).

	Control (*n* = 12)				Increased Diabetes Risk (*n* = 14)				Prediabetes (*n* = 15)				
	Mean	SD	Lower 95% CI	Upper 95% CI	Mean	SD	Lower 95% CI	Upper 95% CI	Mean	SD	Lower 95% CI	Upper 95% CI	*p*
**Mean interstitial glucose (mmol/L)**	5.9	0.5	5.6	6.1	5.9	0.5	5.6	6.1	6.3	0.6	6.0	6.6	0.04
**SD (mmol/L)**	1.0	0.3	0.8	1.1	0.9	0.1	0.8	1.0	1.0	0.2	0.9	1.1	0.55
**CV (%)**	15.2	6.1	11.8	18.7	15.6	2.1	14.5	16.7	15.7	3.2	14.1	17.3	0.95
**TIR (%)**	98	2	97	99	97.9	2.2	96.6	99.1	97.3	4.8	94.6	99.9	0.61
**TAR (%)**	0	1	0	1	0	0	0	0	2	5	0	4	0.22
**TBR (%)**	2	2	1	3	2	2	1	3	1	1	0	1	0.17

CGM-derived metrics include mean interstitial glucose, SD: standard deviation, CV: coefficient of variation, TIR: time in range, TAR: time above range, and TBR: time below range. Data are presented as mean ± standard deviation or median (interquartile range), as appropriate.

## Data Availability

The data presented in this study are available from the corresponding author upon reasonable request. The data are not publicly available due to privacy and ethical restrictions.
